# Clinical reasoning in pharmacy: What do eye movements and verbal protocols tell us about the processing of a case task?

**DOI:** 10.1007/s10459-023-10242-x

**Published:** 2023-06-05

**Authors:** Ilona Södervik, Leena Hanski, Henny P. A. Boshuizen, Nina Katajavuori

**Affiliations:** 1 Centre for University Teaching and Learning (HYPE), Faculty of Educational Sciences, University of Helsinki, Siltavuorenpenger 5B, 00170 Helsinki, Finland; 2 Faculty of Pharmacy, University of Helsinki, Biocenter 2, PO Box 56, 00014 Helsinki, Finland; 3 Faculty of Educational Sciences, Open University of the Netherlands, Valkenburgerweg 177, 6419 AT Heerlen, The Netherlands; 4 Centre for University Teaching and Learning (HYPE), Faculty of Educational Sciences, University of Helsinki, Viikinkaari 9, 00140 Helsinki, Finland

**Keywords:** Case task, Clinical reasoning, Eye-tracking, Pharmacy education, Script activation, Script theory, Script verification

## Abstract

This study investigates pharmacy students’ reasoning while solving a case task concerning an acute patient counselling situation in a pharmacy. Participants’ (*N* = 34) reasoning processes were investigated with written tasks utilizing eye-tracking in combination with verbal protocols. The case was presented in three pages, each page being followed by written questions. Eye movements were recorded during case processing. Success in the task required differentiating the relevant information from the task redundant information, and initial activation of several scripts and verification of the most likely one, when additional information became available. 2nd (*n* = 16) and 3rd (*n* = 18)-year students’ and better and worse succeeding students’ processes were compared. The results showed that only a few 2nd-year students solved the case correctly, whereas almost all of the 3rd-year students were successful. Generally, the average total processing times of the case material did not differ between the groups. However, better-succeeding and 3rd-year students processed the very first task-relevant sentences longer, indicating that they were able to focus on relevant information. Differences in the written answers to the 2nd and 3rd question were significant, whereas differences regarding the first question were not. Thus, eye-tracking seems to be able to capture illness script activation during case processing, but other methods are needed to depict the script verification process. Based on the results, pedagogical suggestions for advancing pharmacy education are discussed.

## Introduction

An objective of universities is to educate future experts who are capable of accurate, flexible and effective information processing both in routine and in complex and varying situations (Ericsson & Kintsch, 1995; Ericsson & Pool, 2016). Although expertise is assumed to be domain-specific to a great extent, similar cognitive processes and transitions seem to be relevant on the way towards expertise across domains (Boshuizen et al., 2020). Case tasks that require authentic-like and discipline-specific reasoning have been recognized as important learning activities for students on their way towards expertise, allowing the intertwining of theoretical knowledge and associated skills (Boshuizen & Schmidt, 2018). One of the most essential expert skills in the health professions is accurate and effective clinical reasoning and decision making. Most research related to reasoning and decision making during expertise development has been conducted in medicine (see e.g. Boshuizen & Schmidt, 1992; de Bruin et al., 2005; Feltovich & Barrows, 1984; Schmidt & Rikers, 2007). However, clinical reasoning also plays an essential role in several other professions, such as in nursing, dentistry and pharmacy (Arisudhana, 2022; Eberhard et al., 2009; Higgs et al., 2018; Walker et al., 2022). In their role of patient counsellor, today’s pharmacists continuously face situations that require clinical reasoning and immediate action.

The purpose of this study was to investigate the reasoning processes of a case task among more and less experienced undergraduate pharmacy students using eye-tracking methodology, written assignments and retrospective stimulated recall interviews. The case handled a relatively common, serious condition in pharmacies that requires identification of the acute condition at hand and immediate reaction accordingly. Pharmacists’ failure in this can have serious consequences for the customer. We hypothesize that a multimethod approach offers interesting insights regarding the case-reasoning processes among and between the student groups providing new knowledge about the role of the illness script approach in pharmacy education.

### Clinical reasoning and script processing in health professions

There has been plenty of research related to cognitive processing in medicine, where diagnostic reasoning naturally is the most critical skill (Boshuizen & Schmidt, 2018; Croskerry, 2009; Norman et al., 2014, 2018). Research on clinical reasoning has taken many different perspectives. These include decision-making theory aiming to optimize the clinical reasoning process (Schwartz & Elstern, 2008) as well as cognitive theories that focus on the interaction between knowledge structure development under the influence of practical experience and its effect on clinical reasoning (e.g., Boshuizen & Schmidt, 2018). The latter approach dates from the 1980s when Feltovich and Barrows (1984) introduced the concept of *illness script.* Illness scripts are chunks of integrated information concerning medical abnormalities in terms of enabling conditions, the fault and consequences (Boshuizen et al., 2012; Custers et al., 1996). Medical students already start to gradually shape professional illness scripts during studies based on clinical reasoning tasks and patient encounters that constantly become refined by experience. Scripts help to find patterns of diseases, filter out irrelevant information, rule out several diseases and construct working diagnoses efficiently and accurately (Monajemi et al., 2012). Although the illness script theory has been developed and the vast majority of studies has been conducted in the medical context, there are studies showing that the theory is also relevant for and can be extended to other health professions as well, such as in nursing (Arisudhana, 2022) and pharmacy (Walker et al., 2022).

The clinical reasoning process starts with script(s) activation, which are then tested, compared and contrasted in the process of script verification (ten Cate & Durning, 2017). In a script verification process the actor attempts to determine whether the activated script or any of the activated scripts adequately fits the findings until all available information that typically becomes gradually available is received (see e.g. Charlin et al., 2007; Södervik et al., 2017). In addition, in real settings, not all received information is equally important, but the decision maker must be able to differentiate the relevant substance and aspects from not-so-relevant, competing noise. Novices and more experienced actors typically differ in their ability to activate and verify appropriate scripts and in their ability to effectively find and focus on the relevant information regarding the case (Monajemi et al., 2012; Schmidt & Rikers, 2007).

### Case tasks and eye-tracking in the investigating reasoning process

Solving case tasks is suggested to facilitate students’ knowledge restructuring process, where conceptual knowledge is rearranged, integrated and reorganized and scripts are created (Boshuizen et al., 2020). Thus, processing and solving authentic, discipline-specific case tasks is assumed to have the potential to reveal students’ level of expertise and to effectively support their learning especially in the early stages of expertise development, when real hands-on problems can still be too demanding and discouraging (see e.g. Boshuizen & Schmidt, 2018; Boshuizen et al., 2020).

Case tasks can be defined as descriptions of specific events or problems that are drawn from the real world of professional practice (Ramaekers et al., 2011). Solving them should require similar mental activities and processes that are faced later on in real work life (Brown et al., 1989). Thus, case tasks activate the meaningful linking of learners’ prior knowledge with the new knowledge and authentic questions deriving from the real-life context (Boshuizen & Schmidt, 1992). However, research has shown that students typically struggle in applying basic knowledge in practical problem-solving situations, such as solving case tasks (e.g. Boshuizen et al., 2020), also in pharmacy context (Persky & Murphy, 2019).

Solving clinical case tasks includes illness script activation and verification processes. Script activation happens fast and autonomously and verbal methods may miss which information triggered it. On the other hand, script activation might be detectable via eye-tracking, which captures the non-conscious processing of the information that receives visual attention (Kok & Jarodzka, 2017). Eye-tracking methodology provides a promising tool to investigate the reasoning and processing of different stimuli and it has been studied from various perspectives, such as via reading strategies and the effect of text structures on eye-movement patterns (Hyönä & Nurminen, 2006; Klusewitz & Lorch, 2000; Wiley & Rayner, 2000) and images or videos as a stimulus in life science contexts (Jaarsma et al., 2014; Jarodzka et al., 2010, 2012). Previous studies suggest that attention allocation differs depending on the level of expertise (Reingold & Sheridan, 2011). Parts of the stimulus considered highly important by the participant seem to receive more visual attention than those which are considered less important (Gegenfurtner et al., 2011; Hyönä & Niemi, 1990). Thereby, attraction of visual attention might be a sign of experienced relevancy to the learner. On the other hand, eye-tracking data do not provide much information regarding the reasons for visual attraction, which is why complementary methods are needed (Kok & Jarodzka, 2017). Hence, in this study, we use written assignments and retrospective stimulated recall interviews to gain a deeper understanding about the reasoning processes.

### Pharmacy discipline as a research context

One commonly perceived megatrend of healthcare systems is the transition from doctor-driven to patient-centred health management. Among other things, this is manifested as people taking an active role in defining their health status and making independent decisions on the use of medicines or other products in the market. Pharmacists involved in patient-centred duties in community pharmacies typically act as critical mediators between the pharmaceutical industry and customers, who may have adopted false or oversimplified information related to drugs and their appropriate use. Inappropriate use of non-prescription medicines is relatively common (Algarni et al., 2021) and adverse drug effects have been reported to account for 6–12% of all hospital admissions among the elderly (Parameswaran Nair et al., 2016). In brief customer encounters, a community pharmacist should identify high-risk patients and recognize conditions where an intervention by health care professional is required to ensure patient wellbeing. Thus, the quality of pharmacy experts’ work drastically contributes to the well-being of human society in numerous levels and hence, fostering the development of expertise is a goal of utmost importance in pharmacy education institutions.

#### Aim

Previous studies have provided interesting insights into reasoning of case type of tasks (see e.g. Boshuizen et al., 2012), but research focusing on the *processes* by which participants use the case description text and graphics while coming to a solution, is scarce. Eye-tracking offers a suitable method for investigating since according to the widely accepted eye–mind hypothesis, the direction of the human gaze is tightly linked to the focus of attention and cognitive processing (Just & Carpenter, 1980). Most of the research concerning clinical reasoning processes has been conducted in the medical field, but there is a need for broadening the scope for other sciences that share similar types of learning challenges. Additionally, despite a large number of studies concerning expertise in comprehension of visualizations, eye-tracking studies using domain-specific, relevant cases as stimuli seem to be relatively scarce.

Based on these premises, the research questions and hypotheses of the study were as follows:

1. What kind of differences in processing exist between 2nd- and 3rd-year pharmacy students and between participants with a correct versus an incorrect or missing conclusion? Previous eye-tracking studies and studies comparing the performance of novices and more experienced actors suggest that more experienced participants and those who diagnose the case correctly will process the case more quickly (e.g. Charlin et al., 2007; Mann et al., 2007; Norman, 2009; Schmidt & Rikers, 2007; Woods et al., 2006). However, other studies report an inverted U-shaped relation between expertise level and several dependent variables—the so-called intermediate effect (Schmidt & Boshuizen, 1993). On the scale of expertise development, pharmacy students are relatively intermediates, which makes it difficult to predict the direction or magnitude of the difference between the groups.
2. Do the processing times and focusing on the task-relevant and task-redundant parts of the case material differ between participants who give a correct versus an incorrect or missing conclusion or between participants with a different level of expertise? Based on previous research, it is hypothesized that those who solve the case correctly will be more effective in directing their attention to the task-relevant areas of the case material and more capable of indicating this in their verbal protocols (Gegenfurtner et al., 2011; Reingold & Sheridan, 2011; Södervik et al., 2017).

## Methods

### Participants and design

The participants (*N* = 38) were native Finnish*-*speaking pharmacy students from the University of Helsinki in spring 2020. The eye-tracking data were poor for four students; hence, a total of 16 s-year students (13 = female; 3 = male) and 18 third-year students (16 = female; 2 = male) were included in the study. The participants represented approximately 11% and 13% of the 2nd- and 3rd-year cohorts respectively. The vast majority of theoretical studies of the study programme were accomplished by both 2nd- and 3rd-year students. However, the second-year students had not yet completed their practical training period which is included in the bachelor’s studies, whereas the third-year students had completed six months of the practical training period in a community or hospital pharmacy before data collection. The third-year students on the other hand, had accomplished almost all their studies in the bachelor programme of pharmacy and were about to graduate as pharmacists in spring 2020. In Finland, pharmacists are graduates of a Bachelor of Pharmacy programme and are eligible to work in responsible duties in community or hospital pharmacies or in drug development. Participation in all studies was voluntary, and informed consent was obtained. Approval of the study was obtained from the ethics review board of the University of Helsinki.

The study was administered in the eye-tracking laboratory, and it consisted of an orientation and a trial phase. The orientation phase included shorter case texts and tasks and its purpose was to familiarize the participants with the test situation. Eye movements were recorded during the case material processing, utilizing with Tobii ProSpectrum (Tobii Technology, Inc., Falls Church, VA). Tobii ProSpectrum allows even large head movements, and as we wanted the reasoning process to be as realistic as possible, no supporting chin rests were used.

At the beginning of data collection, the eye tracker was adjusted accordingly and calibrated, and instructions regarding the orientation and trial phases were given. The participants were told that they would be shown a short case with text and graphics concerning a customer counselling in pharmacy, and after each page, an interspersed slide would ask them to answer written questions without the help of the case material. After the questions had been answered, participants were instructed to continue with the case material by pressing an arrow key on the keyboard. Participants were informed that they should process the material carefully, as it would not be possible to move back to the previous page. There were no time limits, and the participants could move on at their own pace. The session lasted approximately 1 h.

After the trial, a retrospective stimulated recall interview was conducted in which the eye-tracking data were reviewed with the participants and questions were posed about parts of the case material where issues of interest, such as longer fixations or rereading, emerged in the eye movement data. The interviews were video recorded.

### Measures: case and written assignments

The topic of the case, a delayed hypersensitivity reaction, which is a serious adverse effect caused by a commonly used antibiotic was chosen based on the fact that it is relatively common, familiar to students and is a marked reaction that requires acute action from the pharmacist. As in real life, successfully solving the case required differentiating relevant information from the competing noise. The case task (APPENDIX) was presented in three PowerPoint pages and the idea of the case was to simulate the phases of a customer counselling situation in a pharmacy. Dividing the material into three phases allowed us to examine working hypotheses that the students constructed during the process (Schmidt & Mamede, 2015). The case included text and a screenshot from the official drug prescription programme. Parts of the material in each page, such as sentences and sections of the graphics, were semantically categorized into different levels as follows: task-relevant parts including essential information for the solution and task-redundant parts that contained irrelevant or misleading information. Supplementary parts included neutral information, which was not included in the analysis.

The participants were instructed to read the case material carefully so that they understood the content. They were instructed that after each page they would be asked questions that should be answered without the help of the material and that they would not be able to go back to reread the material. It was also emphasized that we were primarily interested in the process, so there was no need to worry about giving uncertain or wrong answers.

**Fig. 1:**
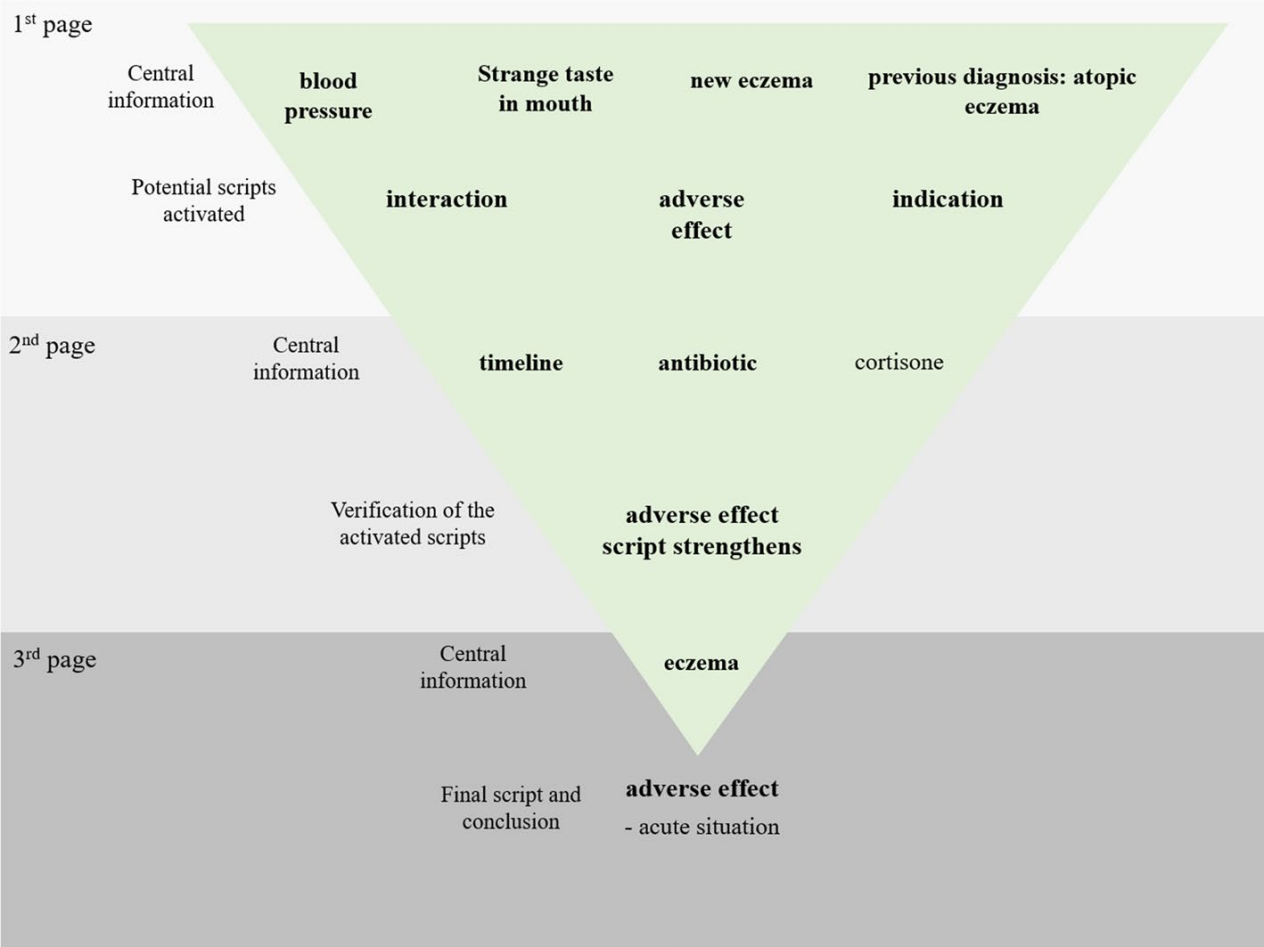
The analysis tool following the structure of the case task. The flow chart illustrates the successful script activation/verification path from the beginning to the final conclusion. The maximum score of the task was 26 (2 points for each element, apart from ‘acute situation’ and ‘cortisone’, which received 1 point)

The case was designed based on the idea that certain working hypotheses i.e. scripts would have been activated at the beginning of the case, and been verified, when new information was provided (Fig. 1). When more information is provided, certain hypotheses stay in the background or become excluded. Finally, there would be a verified, the most likely, script that in this case would be the correct solution (the adverse effect caused by an antibiotic—a condition requiring immediate reaction). The first page provided a spectrum of relevant and redundant initial information related to the client’s acute findings and earlier complaints. This took the form of an encounter where the customer requested that her prescription medication be dispensed. This was expected to trigger scripts related to drug-drug interactions, adverse drug effects and off-label drug use. The second page provided both relevant and redundant information regarding prescriptions and a dispensing list of the customer’s current and earlier medication as an image from the official prescription software. At this stage, verification of the adverse drug reaction script was expected to be more apparent than that of others, owing to a recent prescription of an antibiotic matching a specific set of symptoms presented in the earlier stage. The last page provided both irrelevant information regarding previous findings and more detailed information related to the customer’s acute findings and earlier complaints. This required the student to further compare their script selection with the new information presented before reaching the final conclusion.

After each page, the participants were asked to answer written questions without the help of the case material. The questions asked after each page were as follows: (1) Which aspects did you consider relevant on the page that you have just processed? (2) What is the case about in your opinion? After the last page, there was an additional question: (3) How would you proceed in the situation? The purpose of these questions was to measure participants’ ability to identify relevant aspects from the material, to determine which allowed the participant to make hypotheses about the case and at the end of the case to gain knowledge, whether the participant recognized the serious and acute nature of the case or not. The case task was pilot tested with three pharmacy master’s students, and based on their suggestions, the readability of the case task and the formulation of the questions were improved.

### Data analysis

First, two pharmacists scored the participants’ written answers that were given after each page based on the flow chart model designed by a biologist and two pharmacists (Fig. 1). The correct mentions were scored two points, except for two additional points (‘cortisone’ and ‘acute situation’ in the analysis tool, Fig. 1) that were scored one point each, the maximum score for the case being 26. The final solutions (answers to the questions: ‘What in your opinion is the case about?’ and ‘How would you proceed in the situation?’) made by the participants for the patient case were categorized as either correct or incorrect.

Participants’ case processing data were analysed using TobiiProLab. The analysis of eye-tracking data began with defining the areas of interest (AOIs), the regions in the stimulus from which the authors were interested in gathering data. Processing times for the parts at different semantic levels on each page were calculated together; thus, it was possible to compare the processing times for relevant or irrelevant parts per page. The supplementary parts included neutral information and were excluded from more detailed analyses. A metric of the total visit duration was chosen and it indicates the duration of all visits within an active AOI. IBM SPSS Statistics 25 was used for statistical analyses. Eye movement data were compared between the student groups of correct versus incorrect final conclusions and between the 2nd- and 3rd-year students.

The interview recordings were transcribed word for word and representative answers were presented as examples of the qualitative analyses to gain a better understanding of the processes.

## Results

A total of 20 (59%) out of 34 students gave a correct final conclusion after processing the case (i.e. answers to the questions: ‘What is the case about in your opinion?’ and ‘How would you proceed in the situation?’). The 2nd- and 3rd-year students differed in terms of their success and only four (25%) of the second-year students gave a correct conclusion, whereas almost all (*n* = 16; 89%) of the 3rd-year students succeeded well and provided a correct conclusion. The rest of the students provided an incorrect final conclusion or were unable to provide an answer at the end of the case.

The scores for the written answers did not differ between the groups after processing the first case page. However, those students who solved the case correctly received somewhat higher scores after the second page, (*t*(32) = 1.990, *p* = 0.055) and significantly higher scores after processing the whole case (*t*(32) = 4.891, *p* < 0.000). Furthermore, most of the students (*n* = 14/20, 70%) that solved the case correctly had mentioned the correct working hypothesis (among other possible hypotheses) already after processing the first slide. The reasoning processes remarkably differed between successful and unsuccessful students based on the written answers (see Table 1 for two representative examples). The student who made the correct conclusion had activated two possible scripts after the first page. He/she verified the scripts on the second and third page and provided a correct final conclusion. Whereas the student who made a false conclusion activated only one (false) script of the case, he/she missed the relevant information provided in the forthcoming pages and stayed with the false conclusion. Furthermore, the student with the correct conclusion recognized the risk medication at the early stage of the case, whereas the student with the false conclusion missed this information.

**Table 1:** Examples of a successful (left) and unsuccessful (right) reasoning process in the case

Case material page number	Question	Student A—correct final conclusion	Interpretation	Student B—wrong final conclusion	Interpretation
After the 1st slide	1. Which factors do you find important in this case?	“The symptoms of the customer; urticaria and the bad taste in mouth. Also the blood pressure is satisfactory”	The student immediately brings up the central findings	“The antihypertensive medication and the symptoms which had occurred: the bad taste in the mouth and urticaria”	The student lists the previous medication and findings
	2. What is the case about in your opinion?	“It is probably some kind of side effect either because of the antihypertensive drug or new drug interaction between two or more medicines”	Two scripts activated including the final correct one	“It is probably about the interaction between the antihypertensive drug and some other medication”	Only one script activated
After the 2nd slide	1. Which factors do you find important in this case?	“The recent medicines, for example the amoxicillin which was found”	The student connects a risk medication with the script	“Information about the different medications of the customer”	The student misses the information about the risk medication
	2. What is the case about in your opinion?	“This case is likely to be about an allergic reaction because of the penicillin antibiotic in which urticaria and other skin symptoms may occur”	The student recognizes the correct script and abandons the wrong script	“I think that the symptoms come from the drug interaction between the antihypertensive drug and some other medication”	The student misses the relevant information and continues with a wrong script
After the 3rd slide	1. Which factors do you find important in this case?	“The essential factors are borreliosis and when the symptoms occurred. The diarrhoea is also important”	The student notices additional information	“The use of amoxicillin for the treatment of borreliosis”	The student notices additional information
	2. What is the case about in your opinion?	“I think it is amoxicillin, an allergic reaction to penicillin, and the diarrhoea may also indicate that it is this allergic reaction”	The student verifies the script and ends up with the correct script	“I think it would be possible that those symptoms come from borreliosism, which has not yet been cured”	The student misses the acute situation and ends up with a wrong conclusion
	3. How are you going to proceed with this case?	“I will ask the customer to see the doctor, as the treatment is still going on and it has to be changed”	The student recognizes the serious and acute nature of the case and guides the customer to see the doctor	“I will try to find information from internet whether the antibiotic treatment should be renewed”	The student misses the serious and acute nature of the case

**Fig. 2:**
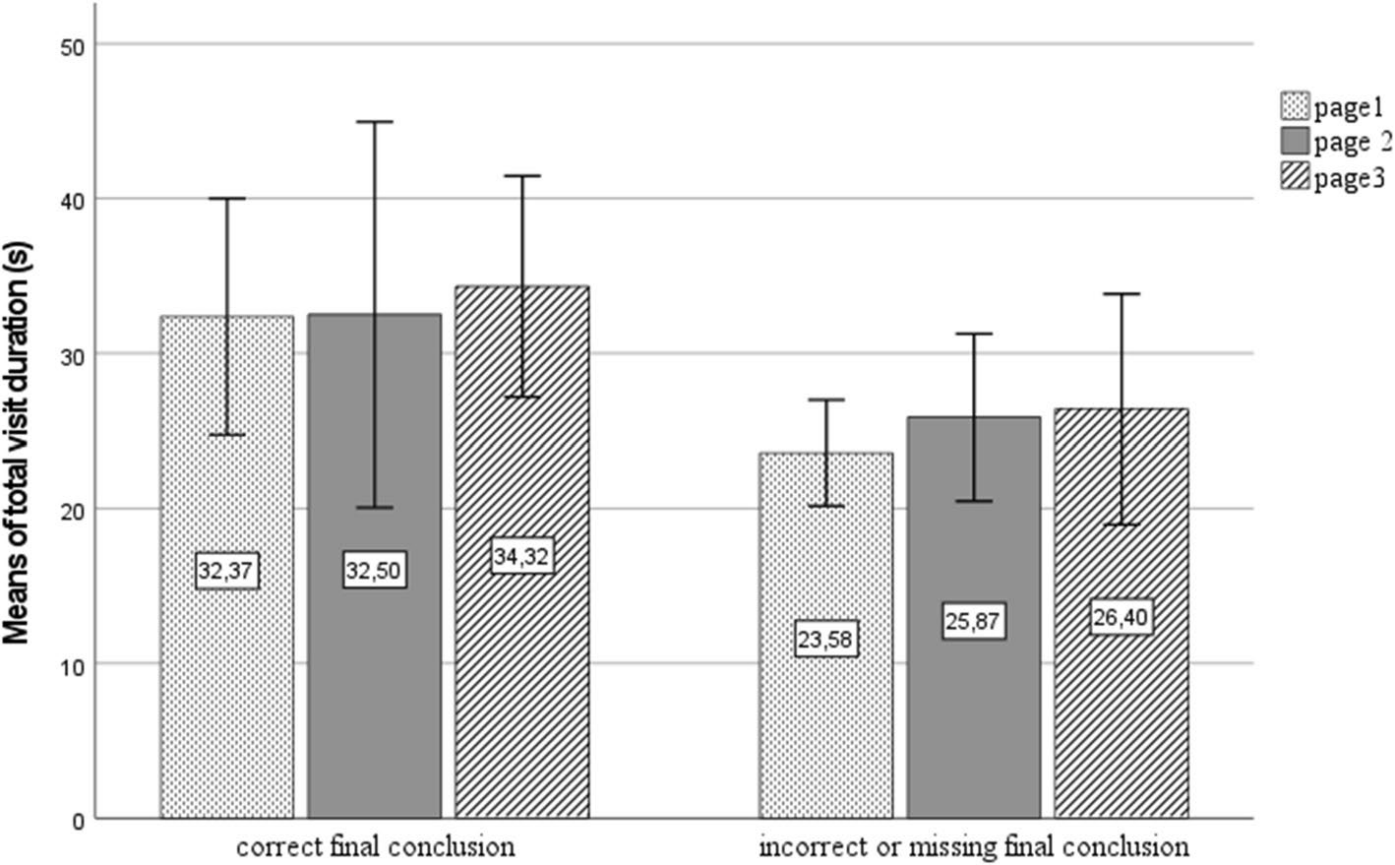
Total visit duration (in seconds) per case page by the groups of students with correct versus incorrect or missing final conclusion (means and standard deviations)

The total average processing times of the case material did not differ significantly between the 2nd- and 3rd-year students or between the students giving a correct versus an incorrect solution. Page-level comparisons revealed significant differences regarding the processing times of the first slide (Fig. 2). Two comparisons were made: the 3rd-year students processed the first page for a significantly longer time than the 2nd-year students (*t* (21.08) = -2.48, *p* = 0.021). Similarly, students who gave a correct final answer took longer to process the first page than the poorer succeeding students (*t*(25.478) = 2.211, *p* = 0.036).

A more detailed sentence-level analysis revealed that 3rd-year students (*t*(19.226) = -2.628, *p* = 0.016) and all students with a correct final solution (*t*(22.708) = 2.187, *p* = 0.039) read the task-relevant sentences (“*At the same time, she has a red raised eczema on her hands and neck. For that purpose, she asks for the cortisone ointment prescribed for her by a doctor”*) of the first slide for a significantly longer time than the 2nd-year or weaker-performing students (Table 2). There were no differences in reading times related to task redundant sentences. Neither were there significant differences in the processing times of semantically different level parts in the following pages of the case. Standard deviations were large, particularly among the 3rd-year students.

**Table 2:** Processing times(s) of different-level sentences between the student groups

	Final conclusion	*N*	*M*	*SD*	Study year	*N*	*M*	*SD*
1st page task-relevant	Incorrect	14	11.06*	3.26	2nd year	16	10.54*	3.02
	Correct	20	17.34*	12.23	3rd year	18	18.50*	12.44
1st page task-redundant	Incorrect	14	9.12	2.58	2nd year	16	9.01	2.56
	Correct	20	11.40	5.95	3rd year	18	11.75	6.14
2nd page task-relevant	Incorrect	14	3.74	2.75	2nd year	16	3.86	2.80
	Correct	20	6.23	6.29	3rd year	18	6.40	6.55
2nd page task-redundant	Incorrect	14	8.67	4.84	2nd year	16	8.61	4.65
	Correct	20	11.52	10.70	3rd year	18	11.90	11.20
3rd page task-relevant	Incorrect	14	6.38	9.99	2nd year	16	6.44	2.86
	Correct	20	8.02	15.41	3rd year	18	8.15	4.42
3rd page task-redundant	Incorrect	14	16.08	2.73	2nd year	16	17.05	9.85
	Correct	20	22.14	4.36	3rd year	18	21.95	16.19

*p < .05

The results of the retrospective stimulated recall interviews show the differences between successful and not successful students regarding their verbal protocols. The successful students, in addition to the fact that they read the relevant sentences longer, were also able to explain verbally in the interview what caught their attention regarding these relevant sentences. This becomes evident in the following two examples (translated from Finnish) from the students with correct final conclusions:

Interviewer*: You have read that sentence* $\left[At\,the\,same\,time\dots eczema\right]$ *particularly carefully. Was there something that you were pondering about related to that?*

Student ID218 (correct final conclusion): *No, it just felt somehow that it was an important sentence. In a sense that is what could be asked there when she tells about the symptoms. It just felt relevant.*

Interviewer: *You have read this *$\left[in\,her\,hands\,and\,neck\right]$* more carefully. Are you able to explain why?*

Student ID215 (correct final conclusion): *It caught my attention that ok, now it’s getting important information... That in her hands and neck there is something that hasn’t been there before.*

Interviewer: *Please summarize which information on that first page made you come up with this working hypothesis?*

Student ID207 (correct final conclusion): *Well, considering that there was not much data available yet, I thought that maybe it was about some side effect caused by some medicine*.

Although there were no statistically significant differences in the processing of the second or third page of the case on the level of eye movements, those students who ended up with a correct solution were better able to focus on relevant aspects of the case material than other students. Students who performed better were able to verify the activated scripts and exclude the wrong ones when more information was provided. These differences in processing among students became evident in the retrospective stimulated recall interview, as can be seen in the following interview citations of one 3rd-year student with the correct final conclusion and one 2nd-year student with an incorrect final conclusion regarding the second page, where the prescribed medication together with dispensing dates were presented:

Interviewer: *You almost immediately started to look at the dispensing dates of the drugs and* *particularly the first preparation (amoxicillin). Are you able to explain why?*

Student ID215 (correct final conclusion): *Well, I knew right away that this is it, because* *penicillin allergy is relatively common. So then I immediately realized it’s about that. This* *was the most important piece of information.*

*…*

Interviewer: *Can you please tell me your conclusion about which information on that page made you to come up with this working hypothesis?*

Student ID215: *It was exactly this amoxicillin, it was right away the most relevant thing in my* *opinion and also the dates. Then also comparison with other drugs if there were also other* *newly prescribed drugs. After that it was possible to exclude those other drugs that most likely* *have not caused any interaction.*

***

Interviewer: *Can you please tell me your conclusion? Please summarize which information on that page made you come up with this working hypothesis?*

Student ID112 (incorrect final conclusion): *Because of those particular drugs, well I thought that one couldn’t say much more about those symptoms, other than based on the previously prescribed medication and if there were some kind of adverse effect... but then there was such medication that it’s very unlikely to cause such (symptoms)... but then again in my opinion actually some might get... But then I thought, well ok, there is this ointment and there was a mention about atopic eczema before, so that’s why I thought that it’s about the reaction of atopic eczema to some irritation.*

The citations presented above show that certain students who succeeded well in the task were able to explain their processing in the interview verbally. However, this high awareness became evident only from the stimulated retrospective recall interviews, where participants’ own eye movements were shown to them and the processing was interviewed by the researcher. On the other hand, the last citation shows that certain students were less successful in focusing on the most relevant aspects of the case, grasping the simplest explanation volunteered by the customer, and failing to recognize the information that required the application of the information provided by the customer with the theoretical, pharmaceutical knowledge learned in the classroom.

## Discussion

The purpose of this study was to establish how second- and third-year pharmacy students solve and process a written case task that required script activation and verification together with filtering the relevant information from the irrelevant. We also investigated how the processing times of the case differed between the participants who gave a correct rather than an incorrect final solution, and how the participant groups differed in making use of task-relevant and task-redundant aspects of the case. Although effective case processing plays an important role in the development of expertise, empirical studies utilizing psychophysiological research methodology in case task processing are still scarce (see, however, Berger, 2019; Södervik et al., 2017; Zumwalt et al., 2015; Vilppu et al., 2016). Better understanding of the cognitive processing of authentic-like case tasks in various disciplines and among actors with different levels of expertise enables advancing the undergraduate instruction in higher education institutions.

The study shows that there are expected differences among pharmacy students in their reasoning skills. 3rd-year students with clinical experience were clearly more successful in the task and almost all of them ended up with a correct final solution, whereas only a few 2nd-year students solved the case correctly. Unlike in several previous studies, the general average total processing times did not differ significantly between the groups (Woods et al., 2006). One reason for the non-significant difference in processing times may be that the difference in expertise level was relatively small between the groups. However, the group of more experienced students and those students who performed better, processed each page for a longer (significant difference only regarding the first page) time than the less advanced or unsuccessful students. This suggests that the participants in this study are still at the increasing slope of the inverted U-shaped relationship between level of medical expertise and clinical case processing, while more advanced or more successful students process the case more slowly due to their broader, but not yet very well organized knowledge structures (Boshuizen & Schmidt, 1992).

Of interest was the fact that the longer processing time of task-relevant sentences in the first case page was related to better success in the case task. Previous research has shown that more experienced actors in a medical context make more use of the very first relevant pieces of information provided in the case material and these items of information work as efficient promoters of script activation for them (Schmidt & Rikers, 2007). We interpret the longer processing time as shown by eye-tracking measures to be an indication of more and better data selection and interpretation, as well as script activation. This result is in line with an earlier study in which the experienced and better-performing actors spent a relatively longer time with the early relevant information investigated via eye movements, though no such difference existed in the later phases of case processing (see Södervik et al., 2017).

Most of the students that solved the case correctly mentioned the correct working hypothesis after the first case page. The results indicate that early activation of the correct script is essential in the reasoning process, as also suggested in previous studies (Charlin et al., 2007; Norman et al., 2018). However, unlike in previous studies conducted in medicine, the sentence that was assumed to act as a script activator, was not a so-called enabling factor, but the symptom (Custers et al., 1996; Södervik et al., 2017). Nevertheless, the scores of written tasks did not differ significantly between the groups of better- or worse-performing students after the first page, indicating that the student groups did not differ in mentioning the relevant findings and set possible hypotheses. Differences in the quality of written answers between the groups of correct and incorrect final conclusions occurred only in the script verification process after the second and third page, when the successful students received significantly higher scores. These results show that early detection of differences in the processing of better- and worse-performing students was not possible with the written assignments alone, for it required a research methodology that captures unconscious processing.

There were eye movement differences in case processing solely in the first page, but differences in the written answers were not significant regarding the first, but regarding both the second and the third pages. This indicates that for pharmacy students at this level of expertise, script verification continues throughout the case until the conclusion. The retrospective stimulated recall interviews showed that students who performed better were also able to verbally explain why they focused on the relevant aspects when processing the case and how their processing progressed when new information became available. The so-called script verification process became evident from certain more experienced students’ verbal protocols, meaning that the students who ended up with a correct solution explained how they tested competing hypotheses and what led them to end up with the correct diagnosis.

### Pedagogical, theoretical, and methodological implications

The results of this study showed that the third-year students succeeded remarkably better in this case task than the second-year students, even though both the second- and third-year students had similar levels of theoretical knowledge that were needed for solving the task. The difference in education relates to practical training, since the third-year students had conducted their six-month practical training period in community or hospital pharmacy, whereas the second-year students were still waiting to enter their practical training period. Thus, it is likely that practical knowledge learnt in pharmacies helped the third-year students to succeed in this case better, as learning in practice fosters the integration of theoretical knowledge to practice and deepens the theoretical understanding of pharmaceutical knowledge (Katajavuori et al., 2006). Research has shown that applying basic knowledge in practical problem-solving situations poses challenges for learners, and therefore learning activities and assignments supporting this, such as case tasks, are important particularly in the early phases of studying (e.g., Boshuizen et al., 2020; Persky & Murphy, 2019).

The results of this study showed that those students who came up with the right solution to this case and recognized the acute situation of the case with the antibiotic adverse effect, noticed immediately at the beginning the significant symptoms of the customer and were able to focus on the relevant information of the case. They were able to focus on the relevant and correct solution when they had tested their assumptions against the information which was given of the case. Thus, when educating pharmacy students on customer counselling it is essential to pay attention to the background information, such as previously prescribed medication, self-care drugs and natural products that the customer uses, from early on in their training at the very beginning and to teach students to test different scripts of cases that occur in the counselling situations. The result that differences between better- and worse-performing students’ information processing were found related to the first page and only that page, highlights the crucial role of early script activation. Reflective discussions of the reasoning process itself, in addition to sharing topic-specific facts, may support the development of case-solving skills and expertise. Equally important, however, is to support students to learn to consciously resist typical biases, such as *premature closure,* which means staying with the first hypothesis and closing the diagnostic process too early without critically testing it in a script-verification process (Charlin et al., 2007; Norman, 2009).

One important model for understanding clinical reasoning and clinical errors is the dual process theory, which identifies two different ways of information processing in any situation. These two approaches have been called intuitive System I and analytical System II decision making processes. System I is related to automatic, intuitive, heuristic and fast processing of information and script-activation as well, whereas System II relates to analytical, systematic, slow, critical processing of information. Hence, System II is associated with script verification processes and accurate diagnosing of atypical cases, where, for example, the customer’s symptoms and findings do not follow the traditional illness script or when differentiating the relevant information from the competing noise is crucial (Croskerry et al., 2014). In this study, System II level processing became evident via written tasks and stimulated recall interviews. The results of this study show that early detecting of differences in processing of successful and unsuccessful students was not possible with the traditional written assignments, but it required research methodology that captures System I level, automated processing. The results of this study show that combining online and offline measures provides interesting data regarding a clinical reasoning of case tasks that would be inaccessible without a process-level research methodology and a mixed methods approach. More research is, however, needed to understand better how script theory and System I/System II theories intertwine.

It is well established that education must focus on the development of adequate knowledge structures, and learning by cases provides a beneficial opportunity to practise using theoretical knowledge and solving authentic-like problems even in the early phases of studies (Boshuizen & Schmidt, 2018). The pedagogical challenge with regard to the development of clinical reasoning skills is that a good performance in one case does not predict success in another (Boshuizen & Schmidt, 2008; Norman et al., 1985). Hence, students need deliberate practice and systematic training to integrate theoretical knowledge with practical experience in order to develop their clinical reasoning skills and illness scripts (Charlin et al., 2007; Feltovich & Barrows, 1984). Practical trainings are therefore crucial for supporting the development of expertise. This was also seen from the results of this study, where those students who had completed six-months of practical training in a community or hospital pharmacy, performed remarkably better in the case task. Nevertheless, working in real conditions with real customers and patients does not provide a forum for the systematic development of clinical reasoning skills. In practical training and workplace learning, supervision and feedback is typically irregular and students are exposed to a random number and different quality of incidents (Norman et al., 2018). Thereby, we see great pedagogical potential in developing an adaptive, process-level formative assessment and feedback system using eye-tracking methodology with different case tasks. This kind of adaptive system could provide personal and on-time feedback for the students during learning and hence allow more systematic training for clinical reasoning.

### Limitations

In order to improve the research design, several issues must be considered in the future. The use of a rather time-consuming eye-tracking method and partially due to the introduction of Covid-19 restrictions resulted in the sample size being rather small and the sample may also be somewhat biased. First, the students who volunteered to participate in the eye-tracking study may be those who are highly motivated and do not struggle with their studies. Additionally, the experimental study design meant that the participants may not have processed the case material exactly as they would normally do, since it was not possible to go back to the previous page. This may have affected participants’ processing patterns. The case topic was chosen based on the importance and familiarity to the students from their previous studies. However, we did not check whether some of the students had, for example, personal experience of the topic. Lastly, the case handled only one topic and further studies with other domains and with different case types are needed.

### Conclusions

This study is one of the first to investigate the processing of a case that includes text and graphics via eye movements among higher education students at different levels of expertise. The results of this study indicate that success in solving a case task is associated with an early focus on relevant information, and eye-tracking captures illness script activation during case processing. Generally, eye-tracking methodology complemented with other research measures appears to have a great deal of potential in evaluating performance, investigating case-reasoning processes and the growth of professional expertise in higher education. This study focused on a case in a pharmacy context, but the results contribute to the general discussion about clinical reasoning that is relevant in several health professions. The transition of the healthcare system also requires changes in higher education and the results of this study advance our understanding of that.

## Appendix

### Instructions for the case

In this case you will be provided some short case material (3 pages) about a customer encounter in a pharmacy. The case is scheduled for June 2019.

Read the material carefully so that you understand the content. After each page, you will be asked questions that should be answered without the help of the material.

Above all, we are interested in the process, so there is no need to worry about giving uncertain or wrong answers.

Please note that you will not be able to go back in the material.

## CASE

### Slide 1

You work as a pharmacist at a pharmacy prescription dispensing point.

A 40-year-old woman arrives as your customer. She has come to get a three-month dose of her antihypertensive drug. The customer/patient says her blood pressure has been good, but recently there has been a strange taste in her mouth. At the same time, it is noticeable that she has red, raised hives on her hands and neck. To treat this, she asks for a cortisone ointment which was previously prescribed for her atopy.

You open the customer’s prescriptions from the pharmacy program.

### Slide 3

After looking at the customer’s purchase history, you continue the conversation with the customer. You ask the customer what condition the amoxicillin was prescribed for. She says it was for Lyme disease, diagnosed ten days ago, with an associated ring-shaped skin reaction that disappeared in a couple of days. However, the rash on her hands and neck appeared the night before, after several asymptomatic days. You ask if the customer has had any other ailments. She says she has suffered from stomach symptoms: her diarrhoea has now lasted for a few days. However, she suspects it is caused by a norovirus which is going around in her child’s kindergarten. You start thinking about the implications of what the customer is saying.
